# Immune Soluble Factors in the Cerebrospinal Fluid of Progressive Multiple Sclerosis Patients Segregate Into Two Groups

**DOI:** 10.3389/fimmu.2021.633167

**Published:** 2021-03-10

**Authors:** Gloria Donninelli, Valeria Studer, Laura Brambilla, Chiara Zecca, Daniele Peluso, Alice Laroni, Daniele Michelis, Renato Mantegazza, Paolo Confalonieri, Elisabetta Volpe

**Affiliations:** ^1^Molecular Neuroimmunology Unit, Istituto di Ricovero e Cura a Carattere Scientifico (IRCCS) Fondazione Santa Lucia, Rome, Italy; ^2^Neuroimmunology and Neuromuscular Diseases Unit, Istituto di Ricovero e Cura a Carattere Scientifico (IRCCS) Istituto Neurologico Carlo Besta, Milan, Italy; ^3^Neurology Department, Martini Hospital, Turin, Italy; ^4^Neurocenter of Southern Switzerland, Ospedale Regionale di Lugano, Lugano, Switzerland; ^5^Faculty of Biomedical Sciences, Università della Svizzera Italiana, Lugano, Switzerland; ^6^Bioinformatics e Biostatistics Unit, Istituto di Ricovero e Cura a Carattere Scientifico (IRCCS) Fondazione Santa Lucia, Rome, Italy; ^7^Department of Neuroscience, Rehabilitation, Opthalmology, Genetics, Maternal and Child Health, University of Genova, Genova, Italy; ^8^Istituto di Ricovero e Cura a Carattere Scientifico (IRCCS) Ospedale Policlinico San Martino, Genova, Italy

**Keywords:** progressive multiple sclerosis, cytokines, chemokines, expanded disability status scale, cerebrospinal fluid

## Abstract

Primary-progressive (PP) and secondary-progressive (SP) multiple sclerosis (MS) are characterized by neurological deficits caused by a permanent neuronal damage, clinically quantified by the expanded disability status scale (EDSS). Neuronal tissue damage is also mediated by immune infiltrates producing soluble factors, such as cytokines and chemokines, which are released in the cerebrospinal fluid (CSF). The mechanisms regulating the production of a soluble factor are not completely defined. Using multiplex bead-based assays, we simultaneously measured 27 immune soluble factors in the CSF collected from 38 patients, 26 with PP-MS and 12 with SP-MS. Then, we performed a correlation matrix of all soluble factors expressed in the CSF. The CSF from patients with PP-MS and SP-MS had similar levels of cytokines and chemokines; however, the stratification of patients according to active or inactive magnetic resonance imaging (MRI) unveils some differences. Correlative studies between soluble factors in the CSF of patients with PP-MS and SP-MS revealed two clusters of immune mediators with pro-inflammatory functions, namely IFN-γ, MCP-1, MIP-1α, MIP-1β, IL-8, IP-10, and TNF-α (group 1), and anti-inflammatory functions, namely IL-9, IL-15, VEGF, and IL-1ra (group 2). However, most of the significant correlations between cytokines of group 1 and of group 2 were lost in patients with more severe disability (EDSS ≥ 4) compared to patients with mild to moderate disability (EDSS < 4). These results suggest a common regulation of cytokines and chemokines belonging to the same group and indicate that, in patients with more severe disability, the production of those factors is less coordinated, possibly due to advanced neurodegenerative mechanisms that interfere with the immune response.

## Introduction

Multiple sclerosis (MS) is an immune-mediated demyelinating disease of the central nervous system (CNS). Approximately 85% of patients with MS show a relapsing-remitting course of the disease (RR-MS), which in 50% of cases turn to a progressive course, termed secondary-progressive MS (SP-MS) (1). The remaining 10–15% of patients suffer from a progressive onset of the disease without relapses, termed primary progressive MS (PP-MS) (2, 3). Both progressive forms of MS are characterized by irreversible neurological decline caused by neuronal and axonal loss, failure of repair mechanisms, and a continuous accumulation of disability that can be measured by the expanded disability status scale (EDSS), a 0.5-point step-based scale with values ranging from 0 to 10 (4–6).

However, a clear comprehension of pathophysiology characterizing progressive MS is still missing. Known pathogenic mechanisms that possibly drive progression include specific immunological processes that are responsible for chronic inflammation at the leptomeningeal space and cerebral blood vessels (5). In the CNS, the main mediators of neuroinflammation are resident innate immune cells, such as microglia (7) and astrocytes (8), as well as monocyte-derived macrophages (9), B cells (10), and T lymphocytes (11), and each of them can promote the pathology by releasing cytokines and chemokines in the cerebrospinal fluid (CSF). Cytokines and chemokines are essential for activating an immune response and play a pivotal role in establishing and maintaining the inflammatory milieu by serving as chemotactic factors, restraining cell-to-cell communication and regulating proliferation and an activation state of immune, as well as neuronal and glial cells (12). Since CSF reflects the specific CNS immune microenvironment, the analysis of its soluble factors can reveal the altered balance between pro-inflammatory and anti-inflammatory cytokines or between neuroprotective and neurotoxic factors involved in the progression of MS. Several studies have investigated the potential role of CSF cytokines and chemokines in MS, with the final aim of better clarifying the pathogenesis of the disease, suggesting biomarkers for diagnosis, prognosis, and eventually predicting the response to therapies (13). However, most of them focused on RR-MS patients, and few studies investigated patients with progressive MS (14–20); moreover, most studies analyzed a limited cohort of patients (16, 21–23), and some studies included patients treated with immunomodulatory therapies that could affect the CSF environment (14, 21, 22, 24). Thus, an extensive analysis of the CSF composition in a large cohort of untreated PP-MS and SP-MS patients is useful. Our study performed on 38 patients with untreated progressive MS revealed that multiple cytokines and chemokines with distinct functions are released in the CSF. Through correlative studies, we highlighted a differential regulation of immune mediators expressed in the CSF of patients with moderate and severe disability. These results shed light on the potential interference between immunological and neurodegenerative mechanisms behind the progressive forms of MS.

## Materials and Methods

### MS Subjects for the CSF Collection

Patients with PP-MS (*n* = 26), SP-MS (*n* = 12), and RR-MS (*n* = 11) according to the established criteria (25) were recruited in the study from the Neurological Institute Carlo Besta (Milan, Italy) and the University of Genova/IRCCS Ospedale Policlinico San Martino Hospital (Genova, Italy). Demographic and clinical data of patients with progressive and RR-MS included in the study are described in Table 1. Approval by the Ethics Committee of the Neurological Institute Carlo Besta (n.40 of April 27, 2017) and Regione Liguria (n.185/2018 of May 28, 2018) and written informed consent forms in accordance with the Declaration of Helsinki from all participants were obtained before study initiation. At the time of the CSF collection, all patients underwent a full neurological assessment and a brain MRI scan; all subjects were positive to the detection of the oligoclonal band and were not treated with any disease modifying drugs or immunosuppressants.

**Table 1:** Demographic and clinical characteristics of progressive and relapsing-remitting MS subjects at the time of CSF collection.

**Parameter**	**PP-MS**	**SP-MS**	**RR-MS**
Number	26	12	11
Gender (male/female)	12/14	4/8	2/9
Age at sample collection (years)	51.3 ± 1.9	48.4 ± 2.7	30.8 ± 2.5
Disease duration (years)	3.8 ± 0.5	10.2 ± 2.4	1.8 ± 0.9
EDSS	3.7 ± 0.3	4.5 ± 0.5	1.8 ± 0.3
MRI (gadolinium+/gadolinium −)	5/21	3/9	–
N. of gadolinium+ lesions	3.5 ± 1.8	1.5 ± 0.5	–

*Data for age at sample collection, disease duration and EDSS are shown as means ± SEM. PP-MS, primary progressive multiple sclerosis; SP-MS, secondary progressive multiple sclerosis; RR-MS, relapsing-remitting multiple sclerosis*.

Demographic and clinical data were derived from medical records. The onset of the MS disease was defined as the first episode of focal neurological dysfunction indicative of MS for patients with SP-MS, or the onset of the progressive symptoms for those affected with PP-MS. Disease duration was estimated as the number of years from the onset to the last assessment of disability. Disability at the time of the CSF collection was evaluated by means of the EDSS (4). Active disease was defined according to the presence of lesions with gadolinium enhancement at baseline MRI.

### MRI

MRI examination (1.5 Tesla) consisted of dual-echo proton density, fast fluid-attenuated inversion recovery, T2-weighted spin echo images, and pre-contrast and post-contrast T1-weighted spin-echo images. All images were acquired in the axial orientation with 3-mm-thick contiguous slices. The presence of gadolinium (0.2 ml/kg i.v.)-enhancing lesions was evaluated by a neuroradiologist who was unaware of the clinical details of patient.

### CSF Cytokine and Chemokine Analysis

The profiles of CSF cytokines and chemokines were analyzed using Bio-Plex multiplex system (Bio-Rad, Hercules, CA, USA) of magnetic bead-based antibody detection kits, following the manufacturer's instructions. Specifically, Bio-Plex Pro Human Cytokine 27-plex (#M50-0KCAF0Y) was used for the detection of the following analytes: interleukin (IL)-1β, IL-1ra, IL-2, IL-4, IL-5, IL-6, IL-7, IL-8, IL-9, IL-10, IL-12p70, IL-13, IL-15, IL-17A, eotaxin, basic fibroblast growth factor (FGF), granulocyte-macrophage colony-stimulating factor (GM-CSF), granulocyte CSF (G-CSF), interferon-gamma (IFN-γ), interferon gamma-induced protein 10 (IP-10), monocyte chemoattractant protein (MCP)-1, macrophage inflammatory protein (MIP)-1α, MIP-1β, platelet-derived growth factor (PDGF)-BB, regulated on activation, normal T cell expressed and secreted (RANTES), tumor necrosis factor-alpha (TNF)-α and vascular endothelial growth factor (VEGF).

The aliquots of CSF (50 μl) were used for analysis, with a minimum of 50 beads per analyte acquired. Each CSF sample was analyzed in duplicate. Median fluorescence intensities were measured using the Luminex 200 System. Standard curves and values were calculated using xPONENT 4.2 software for MAGPIX®. Data were analyzed and reported as concentration readings (pg/ml).

### Statistical Analysis

For pair-wise comparisons of different groups of patients, we used a non-parametric Mann–Whitney *U*-test. Data were presented as mean ± standard error (SEM). The Pearson's correlation coefficient (≥ 0.5 or ≤ 0.5) was considered for statistical analyses of correlations between cytokines. The significance level was *p* ≤ 0.05 without correction for multiple testing.

## Results

### CSF From PP-MS and SP-MS Patients Contains Immune Mediators

**Figure 1:**
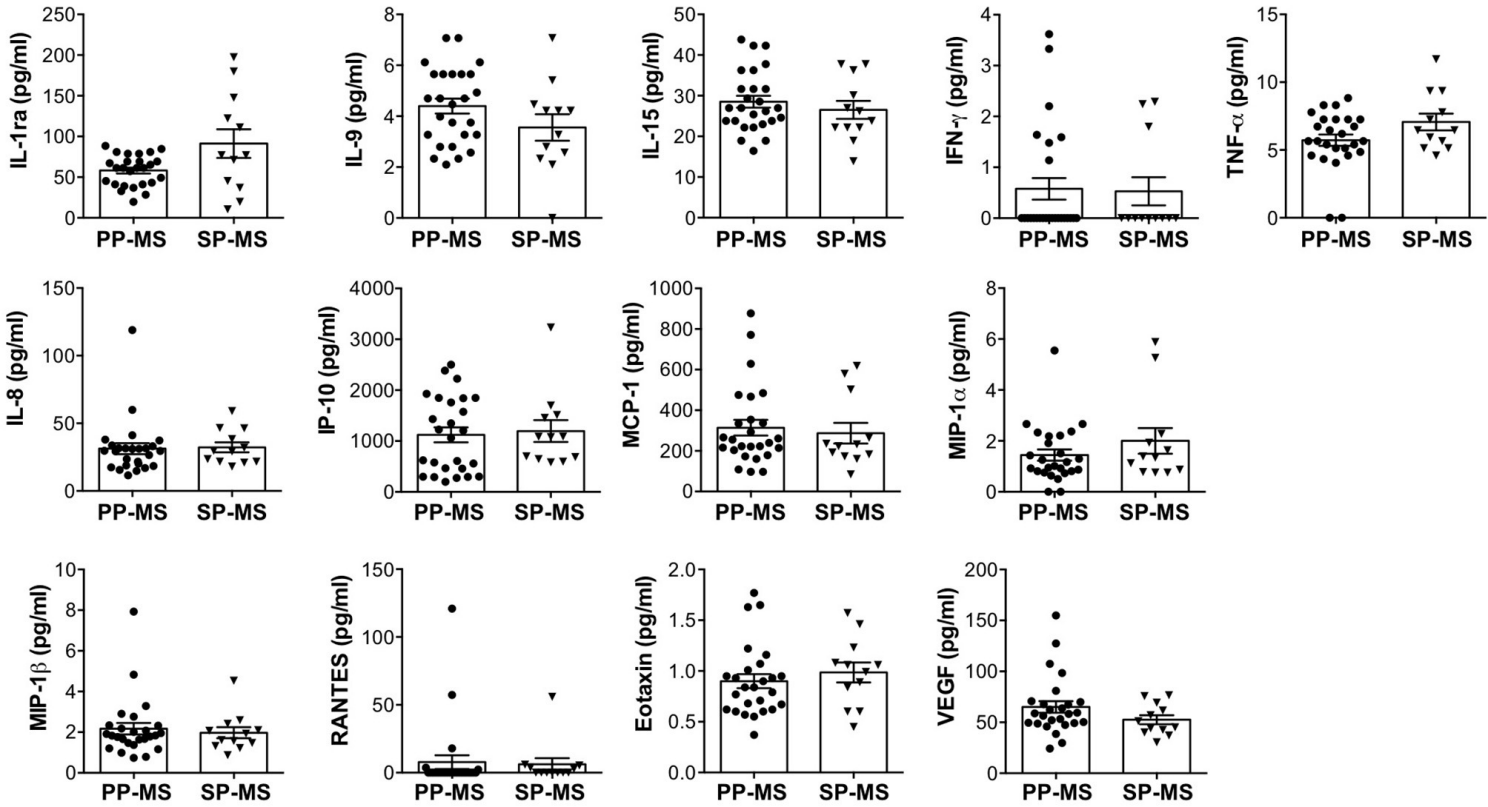
Similar levels of immune soluble factors in the cerebrospinal fluid (CSF) of patients with primary progressive multiple sclerosis (PP-MS) and secondary progressive multiple sclerosis (SP-MS). The levels of interleukin (IL)-1ra, IL-9, IL-15, interferon (IFN)-γ, tumor necrosis factor (TNF)-α, IL-8, interferon-gamma induced protein (IP)-10, monocyte chemoattractant protein (MCP)-1, macrophage inflammatory protein (MIP)-1α, MIP-1β, regulated on the activation, normal T cell expressed and secreted (RANTES), eotaxin, and vascular endothelial growth factor (VEGF) were simultaneously measured in the CSF of PP-MS and SP-MS by multiplex assay. Each CSF sample was analyzed in duplicate and means are represented. Data are reported as mean ± SEM.

In order to define the wide CSF profile of patients with progressive MS, we measured the levels of 27 immune soluble factors in CSF from 26 patients with PP-MS and 12 patients with SP-MS (Table 1), by using a multiplex bead-based assay. We observed that the CSF of both progressive forms of MS contains detectable levels of five cytokines, namely IL-1ra, IL-9, IL-15, TNF-α, and IFN-γ, seven chemokines, namely IL-8, IP-10, MCP-1, MIP-1α, MIP-1 β, RANTES, eotaxin, and the growth factor, VEGF (Figure 1). Interestingly, these soluble factors were found at similar concentrations in the CSF of patients with PP-MS and SP-MS (Figure 1). Additionally, 14 soluble factors (IL-1 β, IL-2, IL-4, IL-5, IL-6, IL-7, IL-10, IL-12p70, IL-13, IL-17A, FGF, GM-CSF, G-CSF, and PDGF-BB), were undetectable in the CSF of patients with both PP-MS and SP-MS (data not shown). These results indicate that a similar pattern of soluble factors characterizes the two progressive forms of MS, suggesting that similar mechanisms regulate the immune response in patients with PP-MS and SP-MS.

**Figure 2:**
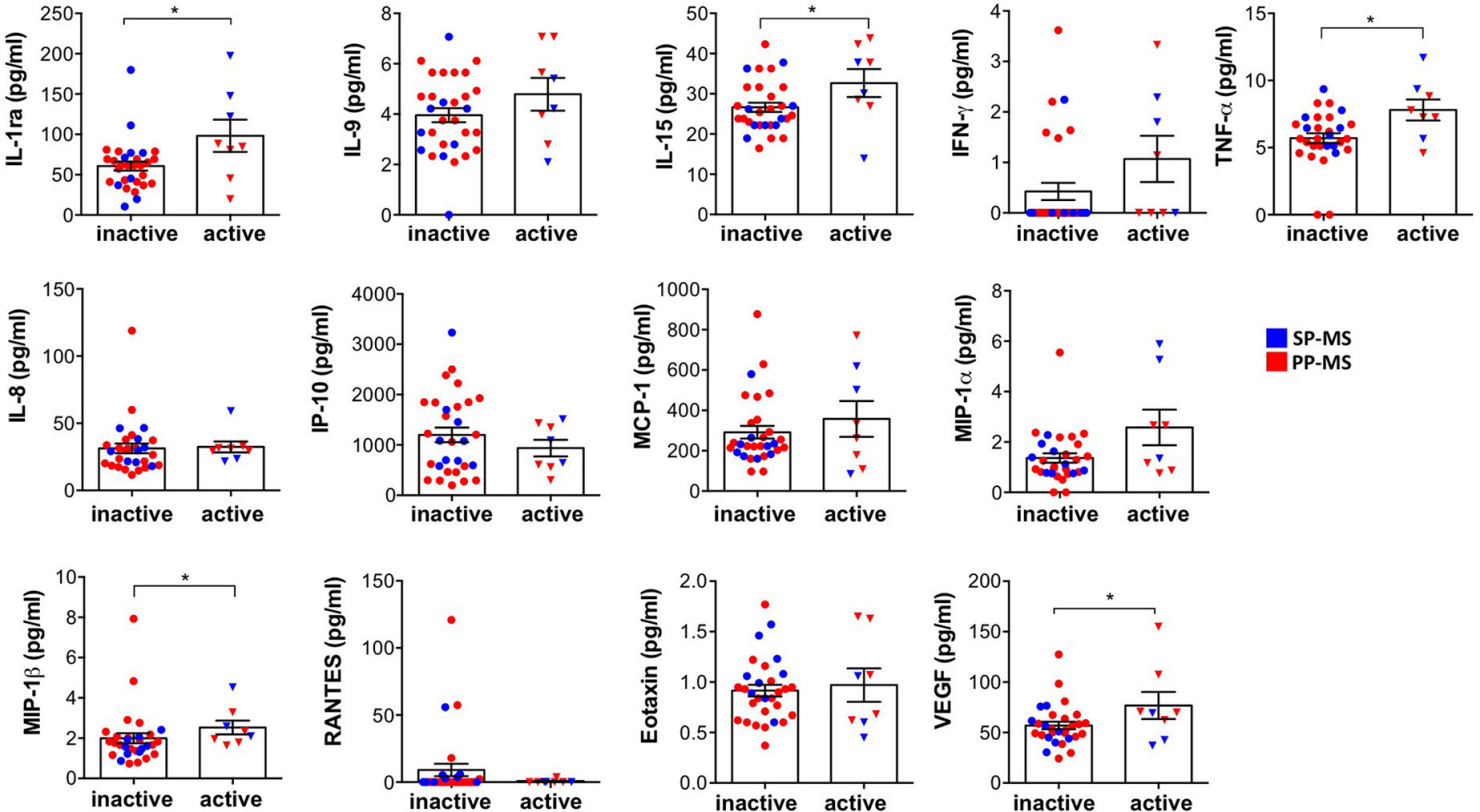
The differential expression of immune soluble factors released in the CSF of patients with progressive MS in active and inactive diseases. The levels of IL-1ra, IL-9, IL-15, IFN-γ, TNF-α, IL-8, IP-10, MCP-1, MIP-1α, MIP-1β, RANTES, eotaxin, and VEGF were measured, by multiplex assay, in the CSF of subjects with progressive MS distinct by the absence (inactive) or the presence (active) of gadolinium detection on MRI. Each CSF sample was analyzed in duplicate and means are represented. Mann–Whitney *U*-test was used to compare different conditions. Data are reported as mean ± SEM. *p < 0.05.

Next, we divided progressive patients into active and inactive MS based on the presence or absence of contrast-enhancing lesions at MRI. We found that the levels of IL-1ra, IL-15, TNF-α, MIP-1β, and VEGF are significantly higher in patients with active MS compared to patients with inactive progressive MS (Figure 2).

### Two Clusters of Immune Soluble Factors Characterize the CSF of Patients With Progressive MS

**Figure 3:**
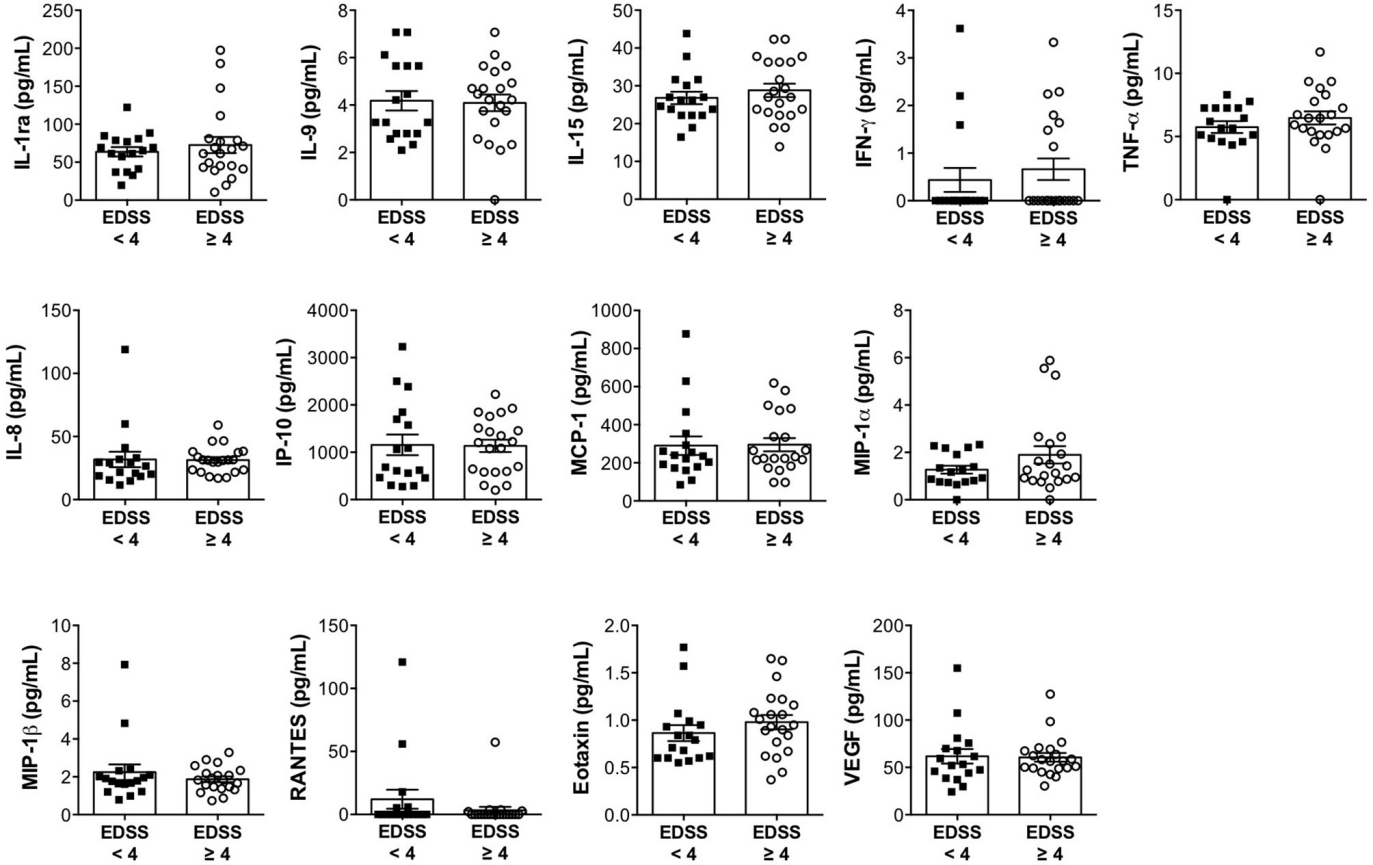
The expression levels of immune soluble factors released in the CSF of progressive MS disease are not associated with expanded disability status scale (EDSS). The levels of IL-1ra, IL-9, IL-15, IFN-γ, TNF-α, IL-8, IP-10, MCP-1, MIP-1α, MIP-1β, RANTES, eotaxin, and VEGF were measured in the CSF of patients with progressive MS with minimal-to-moderate disability (EDSS < 4) or with high disability (EDSS ≥ 4), by multiplex assay. Each CSF sample was analyzed in duplicate and means are represented. Data are reported as mean ± SEM.

In order to investigate whether the expression of immune soluble factors in the CSF of progressive patients is associated with disease severity, we categorized patients in two groups based on disability: mild to moderate (EDSS < 4) and severe (EDSS ≥ 4) disabilities. We obtained two numerically comparable categories of patients, composed of 17 and 21 subjects. The analysis revealed that the levels of CSF soluble factors were not affected by the degree of disability (Figure 3). In fact, IL-1ra, IL-9, IL-15, IFN-γ, TNF-α, IL-8, IP-10, MCP-1, MIP-1α, MIP-1β, RANTES, eotaxin, and VEGF were similarly expressed in patients with mild to moderate or severe disability. Similarly, there was no association between the levels of CSF soluble factors and disease duration (Supplementary Figure 1).

Next, we performed correlative studies between soluble factors expressed in the CSF of patients with progressive MS. This analysis revealed two clusters of molecules significantly correlated in mild to moderate (EDSS < 4) and not in severe (EDSS ≥ 4) disabilities.

**Figure 4:**
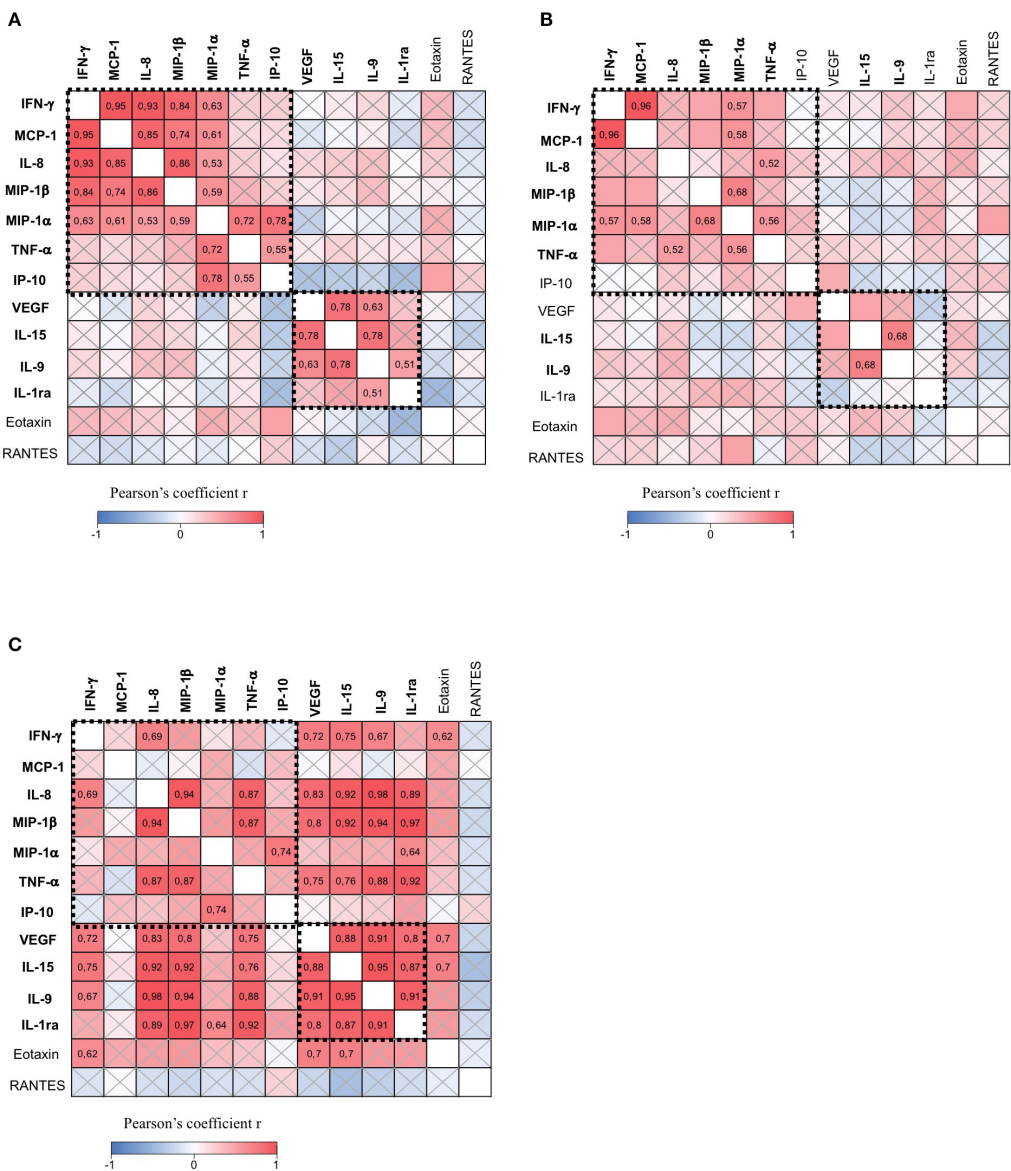
The CSF of patients with progressive MS contains two clusters of immune soluble factors. The correlation matrix among all soluble factors expressed in the CSF of patients with progressive MS with minimal-to-moderate disability (EDSS < 4) (*n* = 17) **(A)**, patients with progressive MS with high disability (EDSS ≥ 4) (*n* = 21) **(B)**, and patients with relapsing-remitting MS (RR-MS) (*n* = 11) **(C)**. Pearson's correlation coefficients *r* ≥ 0.5 or ≤ 0.5 are shown. Crossed squares lack statistical significance (*p* > 0.05). The color in each square indicates the Pearson's correlation coefficient *r* among the variables reported in the two coordinates, as indicated by colored scale bar. The dashed boxes showed two clusters of cytokines and chemokines reciprocally correlated in A: IFN-γ, MCP-1, MIP-1α, MIP-1β, IL-8, IP-10, and TNF-α (group 1), and IL-9, IL-15, VEGF, and IL-1ra (group 2).

Specifically, group 1 contains IFN-γ, MCP-1, IL-8, MIP-1α, MIP-1β, TNF-α, and IP-10, which are positively correlated, and group 2 contains IL-9, IL-15, VEGF, and IL-1ra, which are positively correlated (Figure 4A). Interestingly, soluble factors of group 1 are involved in inflammatory functions, such as the recruitment and activation of T lymphocytes, monocytes, macrophages, and neutrophils, while group 2 contains proteins with anti-inflammatory properties. These results indicate that two classes of soluble factors with opposite functions are simultaneously produced and released in the CSF. Moreover, positive correlations between factors of group 1 suggest that common mechanisms regulate the production of IFN-γ, MCP-1, IL-8, MIP-1α, MIP-1β, TNF-α, and IP-10. Similarly, correlations between factors belonging to group 2 (IL-9, IL-15, VEGF, and IL-1ra) suggest common mechanisms regulating their production.

We observed that the coordinated production of group 1 and group 2 immune mediators is altered in patients with severe disability (EDSS ≥ 4) (Figure 4B) as compared to patients with mild to moderate (EDSS < 4) disability (Figure 4A). In fact, part of the correlations between molecules is lost in patients with higher EDSS, despite similar number of patients in the two groups. For instance, correlations between IL-8 and MCP-1, IFN-γ, MIP-1α, and MIP-1β were not significant, as well as those between IP-10 and MIP1-α, and TNF-α. Other correlations such as those between IFN-γ and MIP-1β or between MCP-1 and MIP-1β were weaker in patients scoring high on the EDSS compared to those scoring low on the EDSS. However, in patients with severe disability, we observed a significant correlation between TNF-α and IL-8 which was not present in patients with mild to moderate disability. Among soluble factors in group 2, we found that IL-9 and IL-15 are the unique cytokines, whose correlation is conserved in progressive MS patients with mild to moderate and severe disabilities. These results suggest that neurodegenerative mechanisms associated with a higher degree of disability may interfere with the global expression of immune soluble factors in the CSF of patients with progressive MS.

Interestingly, we found that soluble factors belonging to group 2 (IL-9, IL-15, VEGF, and IL-1ra) are strongly correlated in patients with RR-MS patients (Figure 4C). Moreover, in those patients, we found that IL-9, IL-15, VEGF, and IL-1ra correlate with soluble factors belonging to group 1, such as IFN-γ, IL-8, MIP-1β, and TNF-α (Figure 4C). These results suggest that, during the onset of the RR-MS disease, there is a simultaneous production of anti-inflammatory and pro-inflammatory soluble factors that could compensate the pathogenic effects of the immune response, thus contrasting the irreversible neurodegeneration typical of the progressive MS forms.

## Discussion

Our study investigates the profile of immune mediators released in the CSF of a large cohort of patients with progressive MS and reveals that CSF microenvironment is characterized by chemokines involved in the recruitment and cytokines involved in the activation of other immune cells. In particular, we found that chemokines, MCP-1 (CCL2), MIP-1 α (CCL3), MIP-1 β (CCL4), RANTES (CCL5), eotaxin (CCL11), IP-10 (CXCL10), and IL-8 (CXCL8), were expressed in the CSF of patients with both PP-MS and SP-MS. In addition, the cytokines IL-15, IL-9, IL-1ra, TNF-α, IFN-γ, and the growth factor, VEGF, were expressed in the CSF of the same patients. These results are consistent with previous data characterizing the immune profile of the CSF of patients with progressive MS (3, 16–21). Moreover, a recent meta-analysis, including 226 studies on patients with progressive and RR-MS, revealed that MIP-1α, eotaxin, IL-8, and IL-15 are significantly increased in the CSF of volunteers with MS compared to the CSF of non-MS (13). However, few studies used a large number of patients with progressive MS, and these validated only the expression of IL-8 (19, 20, 26) and IL-15 (24) as significantly increased compared to controls. Studying the CSF from 38 patients with progressive MS, we found the expression of 13 immune mediators, including IL-8 and IL-15.

Our analysis revealed that PP-MS and SP-MS do not differ in the composition of CSF environment, whereas MRI disease activity was associated with a weak increase of IL-15, VEGF, IL-1ra, MIP-1β, and TNF-α in progressive MS. Interestingly, we further investigated the CSF of those patients by correlative studies and we found that two groups of soluble factors are reciprocally correlated. Group 1 contains IFN-γ, MCP-1, IL-8, MIP-1β, MIP-1α, TNF-α, and IP-10, while group 2 contains VEGF, IL-15, IL-9, and IL-1ra. Notably, group 1 contains chemokines favoring the infiltration and activation of T lymphocytes, neutrophils, monocytes, and macrophages but also of resident CNS cells, thus ultimately contributing to a compartmentalized inflammation in the CNS (22, 23, 27, 28). Moreover, two cytokines of group 1, IFN-γ and TNF-α, also associated with the CSF of patients with progressive MS with a high degree of meningeal inflammation and a high number of cortical lesions (29), play a crucial role in enhancing excitatory synaptic transmission (30, 31), thus favoring neurodegeneration (17). On the other side, group 2 contains soluble factors with potential protective functions. In fact, IL-1ra is a known anti-inflammatory endogenous molecule acting as a competitive inhibitor of IL-1β (32) and demonstrated as effector molecule in reducing disease severity in the murine models of MS (33–35). IL-1ra significantly correlates with IL-9, which has been recently associated with reduced inflammation and reduced neurodegeneration in MS (36, 37). Additionally, we observed a strong correlation between IL-9 and IL-15, whose role in MS is still unclear but could exert a protective role by attenuating the cytotoxicity of CD8-positive T cells (38). Interestingly, the correlation between IL-9 and IL-15 was already reported in patients with RR-MS, and this association was related to the increased levels of both cytokines in patients receiving prednisolone treatment than those without immunotherapy during the relapse (22). These results indicate that IL-9 and IL-15 could share a common mechanism of production and that they are induced by immune-suppressive therapies.

Another factor associated with IL-9 and IL-15 in group 2 is VEGF, which is produced not only by immune cells but also by endothelial cells, astrocytes, and neurons, and that acts as neuroprotective agent for neurons and neural progenitors in the late MS phase, such as the progressive forms of MS (39). Thus, the presence of a specific cluster of immune soluble factors in the CSF composed by potential protective factors as IL-9, IL-1ra, IL-15, and VEGF in progressive MS could represent an attempt of the immune system to counteract the pro-inflammatory environment regulated by the factors of group 1. Importantly, the majority of the correlations between factors belonging to group 1 and those of group 2 were lost in patients characterized by more severe disability (EDSS ≥ 4) compared to patients with mild to moderate disability (EDSS < 4), suggesting that the pro-inflammatory and anti-inflammatory networks generated by the immune system in the CNS is affected by advanced neurodegenerative mechanisms typical of high disability. Indeed, in patients with low disability, the coordinated production of soluble factors with pro-inflammatory and anti-inflammatory functions generates a balanced environment. In contrast, in patients with high disability, the uncoupled production of pro-inflammatory and anti-inflammatory immune mediators in the CSF might interfere with proper resolution of inflammation.

Finally, using a classical approach, this study revealed that the CSF from patients with PP-MS and SP-MS does not significantly differ. Correlative studies, which reflect the coordinated and simultaneous expression of molecules, indicate that the global pattern of immune soluble factors released in the CSF of patients with progressive MS differ according to the level of disability. Moreover, our study revealed that in patients with RR-MS, where the level of disability is consistently lower in comparison to progressive MS, the expression of immune soluble factors in the CSF is even more different. In fact, we found a coordinated expression of molecules with both pro-inflammatory and anti-inflammatory properties that could contribute to the immune compensatory mechanisms and to a better clinical prognosis.

These matrices generated by correlative studies could be a useful tool to globally explore the CSF environment, at different disease stages, during disease activity or disease-modifying therapies, or in other neurodegenerative diseases.

## Data Availability Statement

The raw data supporting the conclusions of this article will be made available by the authors, without undue reservation.

## Ethics Statement

The studies involving human participants were reviewed and approved by Ethical committee of the Istituto Neurologico Carlo Besta and Regione Liguria. The patients/participants provided their written informed consent to participate in this study.

## Author's Note

Multiple sclerosis (MS) is an immune-mediated disease of the central nervous system (CNS). Approximately 85% of MS patients show a relapsing-remitting disease that may turn to a progressive course termed secondary progressive MS (SP-MS). The 10–15% of patients suffer from a primary progressive MS (PP-MS). Both progressive forms of MS are characterized by accumulation of disability measured by the Expanded Disability Status Scale (EDSS). A complex interaction between immune cells and CNS resident cells is involved in the pathology of MS. However, the mechanisms regulating MS progression are still unknown. Since immune soluble factors released in the cerebrospinal fluid (CSF) are critical mediators of the intercellular communication between resident and infiltrating cells in the CNS, their characterization could help understanding the mechanisms regulating the disease. Our study provides a comprehensive characterization of immune soluble factors expressed in progressive MS patients. We found that soluble factors clustered in two groups with pro-inflammatory and anti-inflammatory functions, respectively, and such segregation is weaker in patients with high EDSS (≥4)than in those with mild to moderate EDSS (<4). These data indicate that in patients with more severe disability the production of soluble factors is less coordinated, likely due to advanced neurodegenerative mechanisms that interfere with the immune response.

## Author Contributions

GD performed research, analyzed data, and drafted the paper. VS and LB provided samples and the clinical data of patients with progressive MS and performed research. CZ provided samples and clinical data of patients with progressive MS. DP performed statistical analysis. AL and DM provided samples and clinical data of patients with RR-MS. RM and PC coordinated the recruitment of patients with progressive MS. EV designed research, analyzed data, and wrote the paper. All authors contributed to the article and approved the submitted version.

## Conflict of Interest

The authors declare that the research was conducted in the absence of any commercial or financial relationships that could be construed as a potential conflict of interest.

## Supplementary Material

Supplementary Figure 1 The expression levels of immune soluble factors released in the CSF of progressive MS patients are not associated with disease duration. Levels of soluble factors in the CSF of patients with progressive MS were correlated to their disease duration (years) at the time of CSF sample collection, using the Pearson's correlation coefficient. None of the correlations were statistically significant.

## Abbreviations

MS: multiple sclerosis
CNS: central nervous system
RR: relapsing remitting
PP: primary progressive
SP: secondary progressive
MRI: magnetic resonance imaging
EDSS: expanded disability status scale
CSF: cerebrospinal fluid
IL: interleukin
IFN: interferon
TNF: tumor necrosis factor
MIP: macrophage inflammatory protein
MCP: monocyte chemoattractant protein
GM-CSF: granulocyte-macrophage colony-stimulating factor
CCL: chemokine (C-C motif) ligand
CXCL: chemokine (C-X-C motif) ligand
FGF: fibroblast growth factor
PDGF: platelet-derived growth factor
VEGF: vascular endothelial growth factor
RANTES: regulated on the activation, normal T cell expressed and secreted
IP: interferon gamma-induced protein.
