# Supplementary figures and images for: Immune Soluble Factors in the Cerebrospinal Fluid of Progressive Multiple Sclerosis Patients Segregate Into Two Groups
Source: Front Immunol. 2021 Mar 10;12:633167. doi: 10.3389/fimmu.2021.633167 (PMC7988186; doi:10.3389/fimmu.2021.633167)

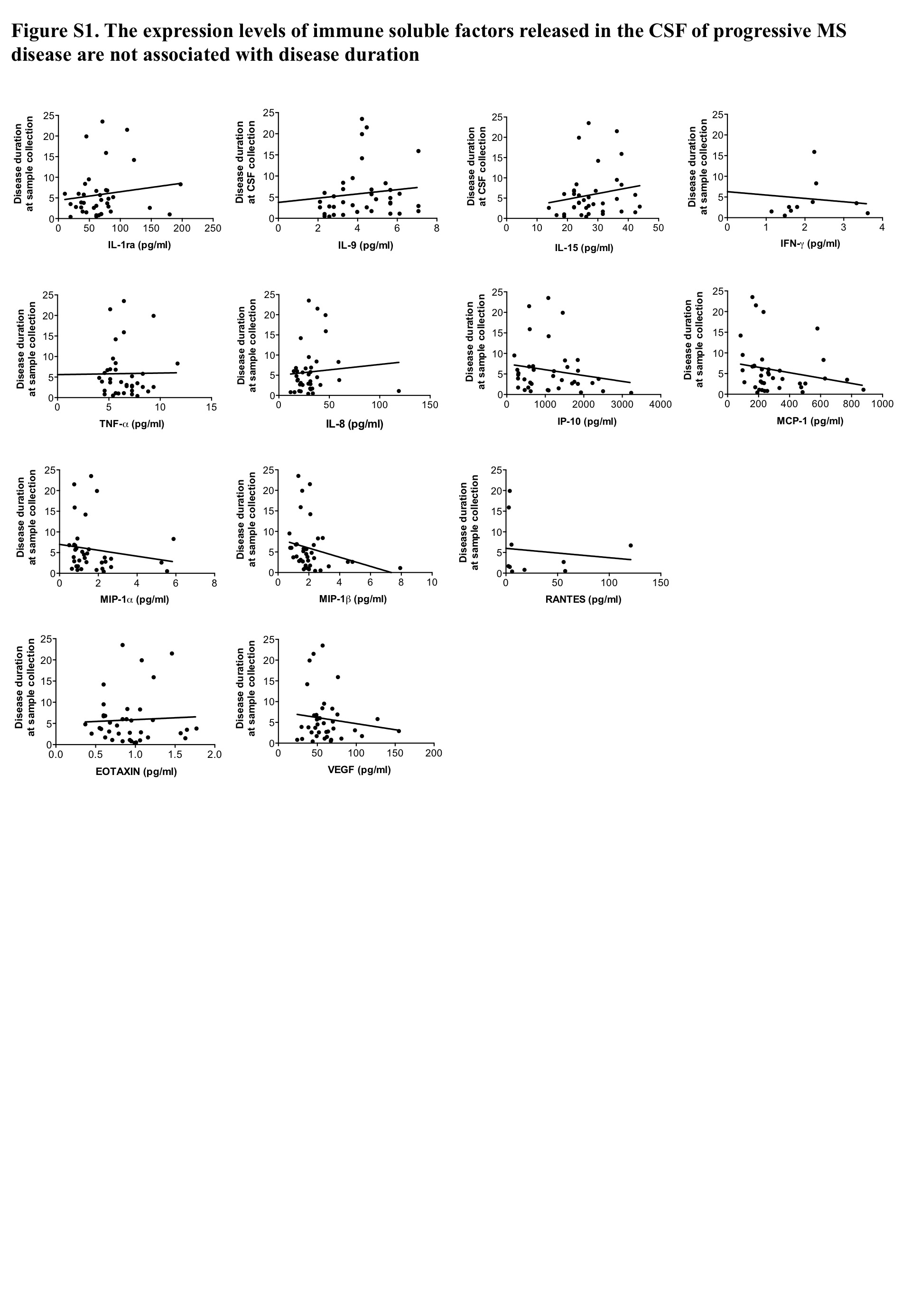

Supplement: Supplementary Figure 1 — The expression levels of immune soluble factors released in the CSF of progressive MS patients are not associated with disease duration. Levels of soluble factors in the CSF of patients with progressive MS were correlated to their disease duration (years) at the time of CSF sample collection, using the Pearson's correlation coefficient. None of the correlations were statistically significant. [file Image_1.JPEG]
